# Supplementary material for: The interactome of intact mitochondria by cross-linking mass spectrometry provides evidence for coexisting respiratory supercomplexes
Source: Mol Cell Proteomics. 2017 Dec 8;17(2):216–32. doi: 10.1074/mcp.RA117.000470 (PMC5795388; doi:10.1074/mcp.RA117.000470)
Supplement: Supplemental Data [file supp_RA117.000470_133922_0_supp_23978_fzffwf.zip › spectra_annotation/mito_DR_spectra_annotation/466-1-19-1-4-1.pdf]

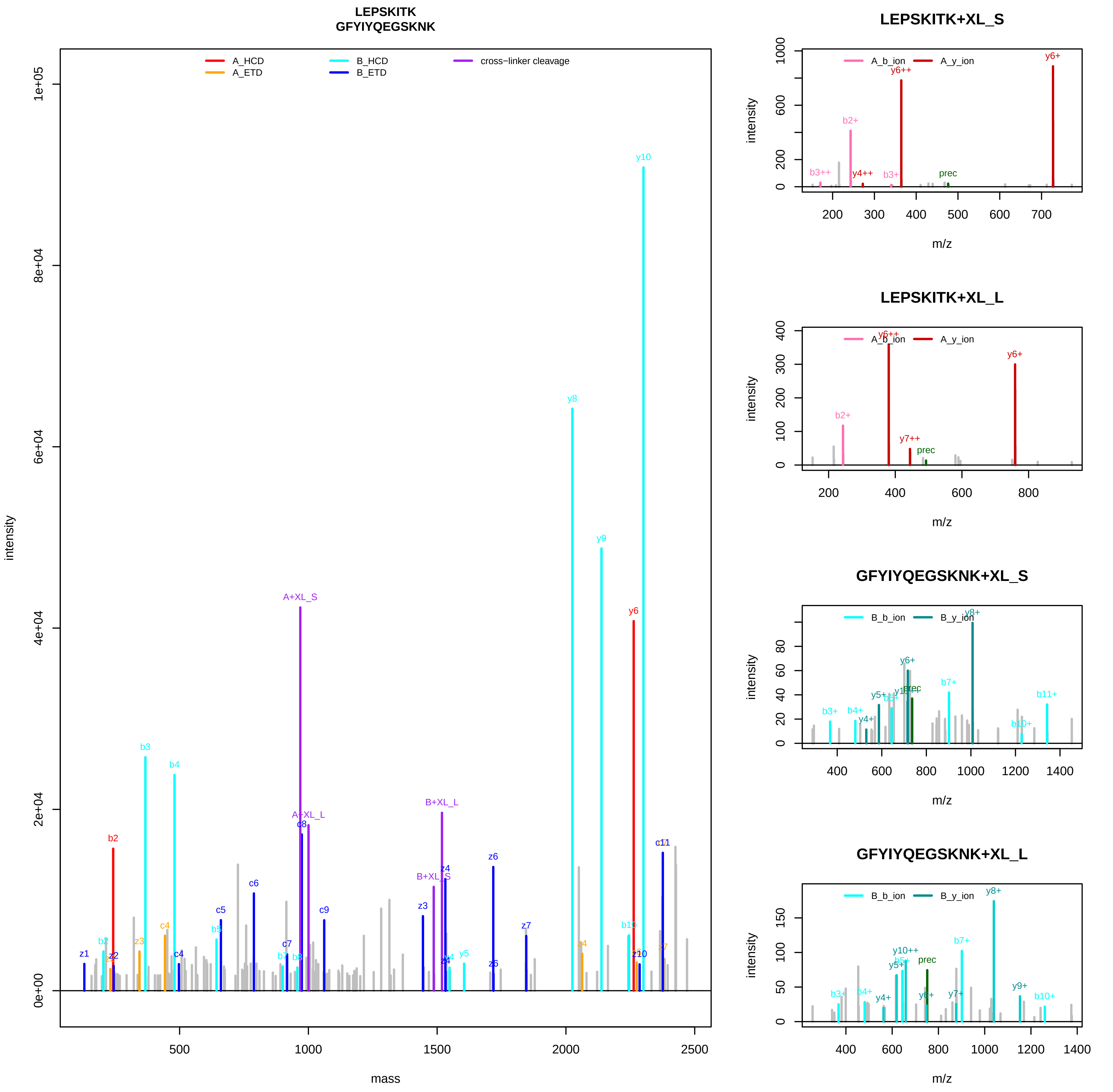

LEPSKITK
GFYIYQEGSKNK
A_HCD
A_ETD
B_HCD
B_ETD
cross−linker cleavage
intensity
mass
0e+00
2e+04
4e+04
6e+04
8e+04
1e+05
500
1000
1500
2000
2500
y10
y8
y9
y6
A+XL_S
A+XL_L
B+XL_L
B+XL_S
b2
b3
b4
b5
c4
c5
c6
c7
c8
c9
c11
z1
z2
z3
z4
z6
z7
z10
y5
b10
LEPSKITK+XL_S
LEPSKITK+XL_L
GFYIYQEGSKNK+XL_S
GFYIYQEGSKNK+XL_L
A_b_ion
A_y_ion
B_b_ion
B_y_ion
y6+
y6++
b2+
b3++
y4++
b3+
y7++
prec
b3+
b4+
y4+
y5+
b7+
y8+
y6+
b11+
b10+
y10++
y9+
m/z
